# Primary Laser Photocoagulation for Giant Retinal Tears Without Retinal Detachment: Two Case Reports and Review of the Literature

**DOI:** 10.7759/cureus.111011

**Published:** 2026-06-17

**Authors:** Salma Orfi, Younes Tlemcani, Sarah Belghmaidi, Abdeljalil Moutaouakil

**Affiliations:** 1 Ophthalmology, Centre Hospitalier Universitaire, Mohammed VI Hospital, Faculty of Medicine and Pharmacy, Cadi Ayyad University, Marrakech, MAR

**Keywords:** argon laser photocoagulation, atypical retinal tear, giant retinal tear, laser retinopexy, peripheral laser cerclage

## Abstract

Giant retinal tears are full-thickness retinal breaks extending 90° or more circumferentially and are typically associated with a high risk of rhegmatogenous retinal detachment and proliferative vitreoretinopathy. Surgical intervention remains the standard treatment in most cases. We report two cases of giant retinal tears without retinal detachment treated with primary argon laser photocoagulation. A 56-year-old man presented with a superotemporal retinal tear in the left eye extending from 12 to 4 o'clock, while a 56-year-old woman presented with a temporal retinal tear in the right eye extending from 7 to 11 o'clock. In both cases, the retina remained attached, with no subretinal fluid or macular involvement. Treatment consisted of barrier laser photocoagulation around the tear combined with 360° peripheral laser cerclage and prophylactic laser treatment of the fellow eye. At 12 and 18 months of follow-up, respectively, both patients maintained a best-corrected visual acuity with stable retinal attachment, well-formed laser scars, and no evidence of proliferative vitreoretinopathy or other complications. To our knowledge, this is among the few reported cases of GRT without retinal detachment successfully managed with laser photocoagulation alone combined with 360° peripheral laser cerclage.

## Introduction

Giant retinal tears (GRTs) are defined as a full-thickness neurosensory retinal break extending circumferentially for three or more clock hours (≥90°), typically but not exclusively occurring in the presence of a posterior vitreous detachment (PVD) [1,2]. GRTs are uncommon, with a reported incidence of approximately 0.09 cases per 100,000 persons per year [3]. Their clinical significance lies in their strong association with rapidly progressive rhegmatogenous retinal detachment (RRD) and proliferative vitreoretinopathy (PVR) [1,4].

Surgical management remains the standard of care for most GRT-associated detachments and typically involves pars plana vitrectomy (PPV) with perfluorocarbon liquids, long-acting gas or silicone oil tamponade, with or without scleral buckle [2,5]. These achieve high initial anatomical success rates of 89% to 94% [2,4]. However, recurrent retinal detachment (RD) has been reported in up to 45% of cases, most commonly due to PVR or the development of new retinal breaks [4]. In addition, invasive surgical intervention carries significant ocular morbidity, including retinal slippage, additional break formation, cataract progression, elevated intraocular pressure, hypotony, phthisis bulbi, and potential perfluorocarbon liquid toxicity [2].

While most reported GRTs present with established RD requiring surgical intervention, identification of GRT prior to the development of clinically significant detachment is rare, and only a limited number of case reports have described successful management using primary laser photocoagulation alone [2,5,6].

In this report, we describe two cases of conservative management of GRTs with primary laser photocoagulation combined with 360° peripheral laser cerclage and prophylactic treatment of the fellow eye. We additionally provide a focused review of the literature. Given the limited evidence available for these uncommon presentations, we aim to add to the existing literature supporting conservative management in carefully selected patients. Our cases also highlight the potential role of adjunctive 360° peripheral laser cerclage in promoting retinal stability and reducing the risk of subsequent retinal detachment.

## Case presentation

This prospective case series was conducted in the ophthalmology department at the Mohammed VI university hospital in Marrakech over a five-year period. During this interval, two consecutive patients presenting with GRTs without associated retinal detachment were identified and managed with primary laser photocoagulation. Clinical data, imaging findings, treatment details, and follow-up outcomes were prospectively collected. Both patients underwent regular follow-up examinations to assess retinal attachment status, visual outcomes, and treatment-related complications.

Case 1

**Figure 1:**
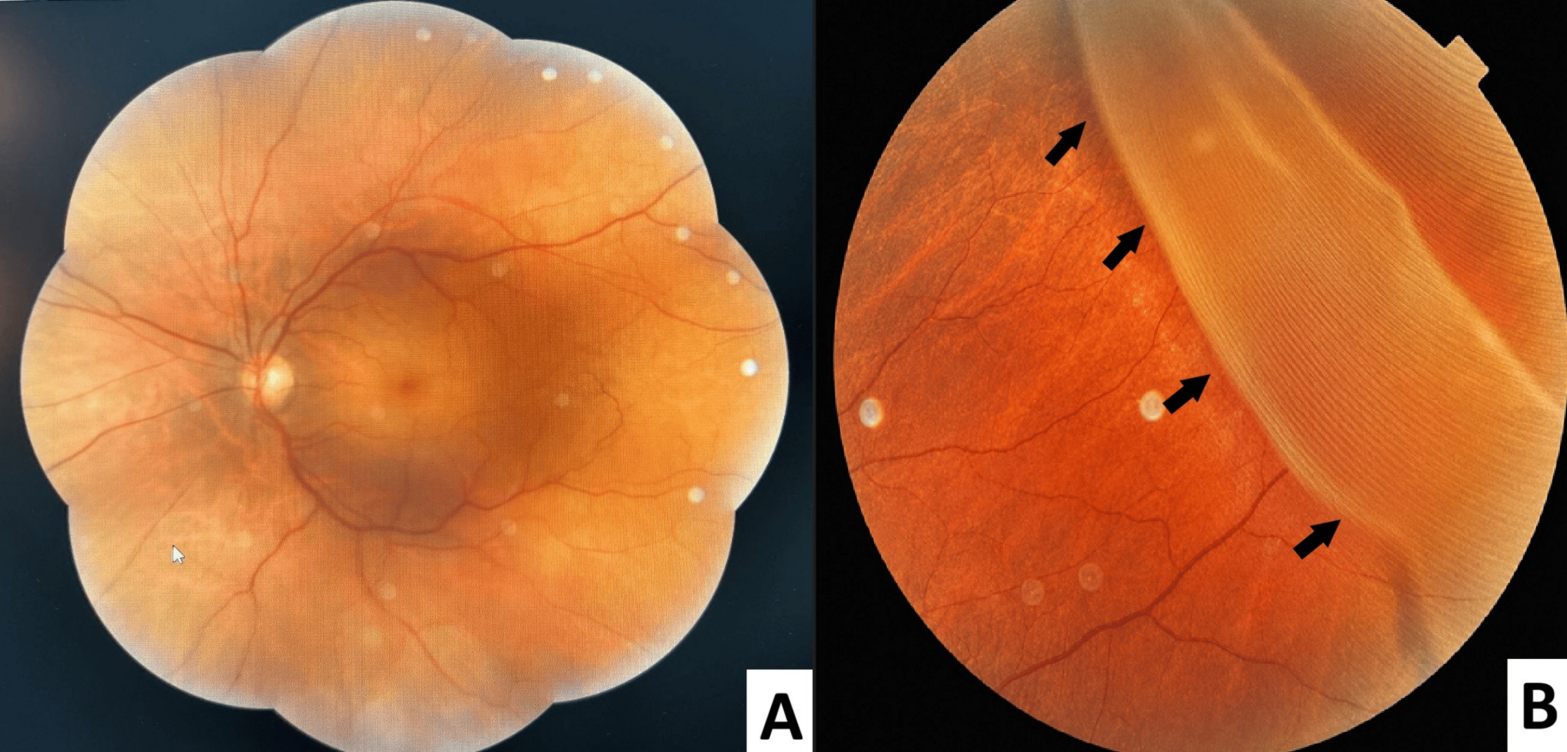
Fundus photograph prior to laser photocoagulation A: Wide-field mosaic fundus imaging demonstrates a fully attached retina, with no evidence of retinal detachment up to the equator. B: Fundus photograph of the extreme superotemporal retinal periphery demonstrating a giant retinal tear with an inverted retinal flap (black arrows), without evidence of subretinal fluid

A 56-year-old man with no significant past medical or ocular history presented with a one-week onset of blurry black pinpoint vision associated with photopsias in the left eye. On examination, his best-corrected visual acuity (BCVA) was 10/10. The anterior segment examination was unremarkable with a clear crystalline lens. The fundus examination revealed a GRT extending from 12 to 4 o’clock in the superotemporal quadrant with a rolled posterior edge and no subretinal fluid was detected. The retina remained fully attached with no macular involvement (Figure 1). Examination of the right eye showed no abnormalities.

After a thorough discussion of risks and complications, and given the absence of retinal detachment, a conservative approach was adopted. The patient underwent argon laser photocoagulation, which consisted of barrier laser around the tear, 360° peripheral laser cerclage and a prophylactic 360° laser cerclage of the right eye. Laser parameters were as follows: spot size: 200 µm, power: 150 mW and duration: 150 ms.

**Figure 2:**
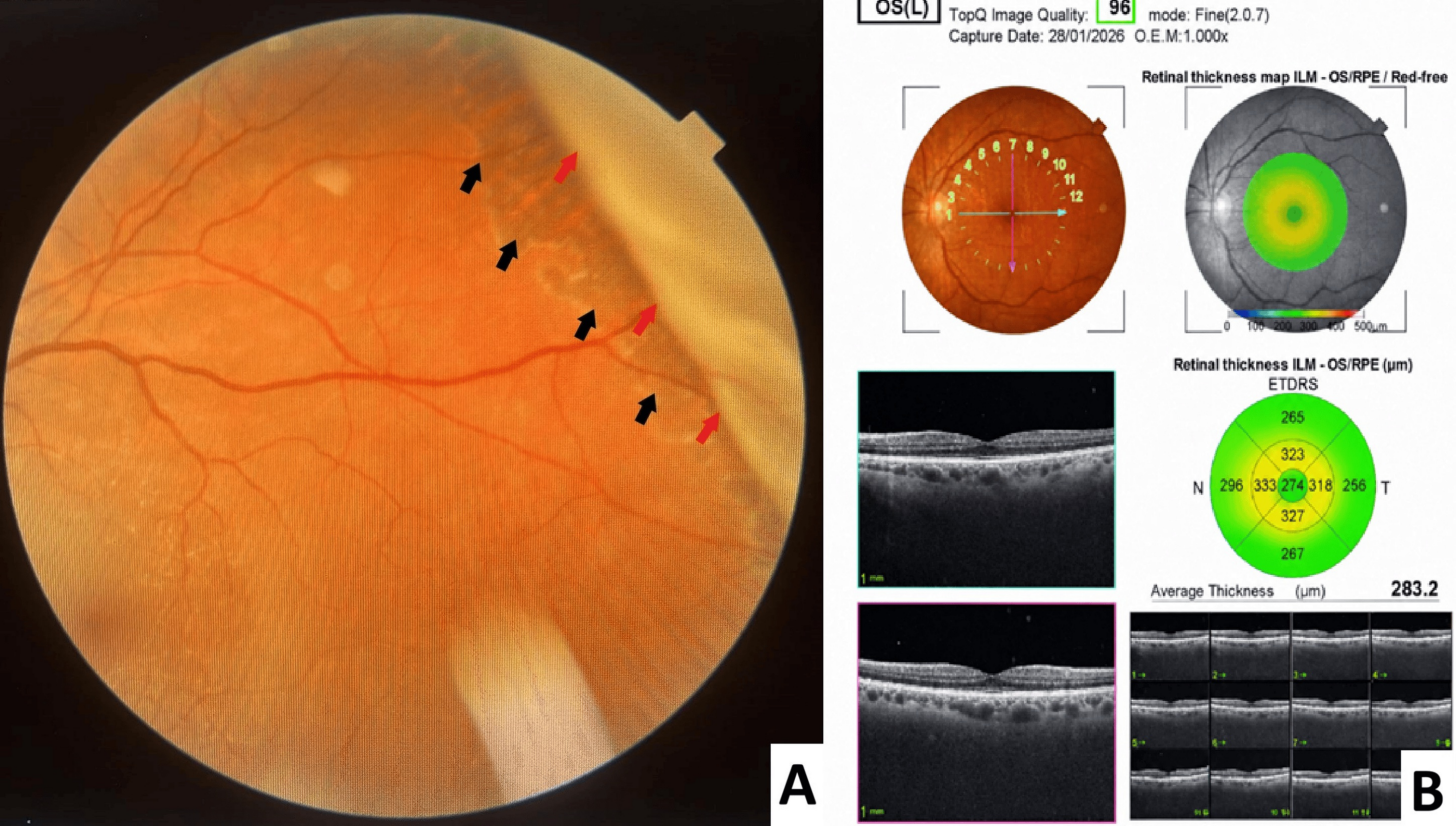
Follow-up fundus photography and optical coherence tomography (OCT) A: Fundus photograph showing a well-defined retinopexy scar (black arrows) and stable retinal tear (red arrows). B: Normal macular OCT with preserved retinal architecture.

At 12-month follow-up, BCVA remained stable at 10/10. Funduscopic examination demonstrated a flat and attached retina, with well-formed laser scars and a normal macular profile. No complications, including proliferative vitreoretinopathy, were observed (Figure 2).

Case 2

**Figure 3:**
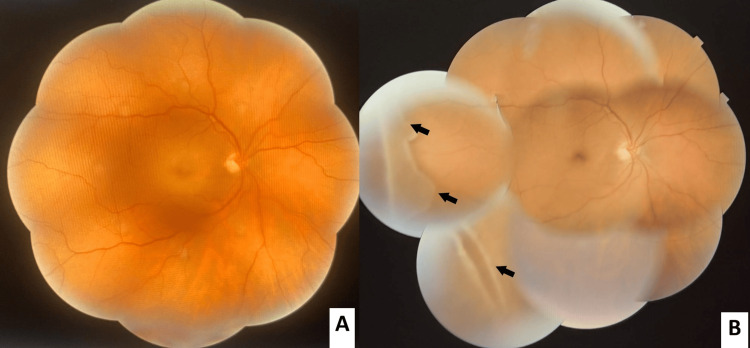
Fundus photography of the second patient prior to laser photocoagulation A: Wide-field mosaic fundus imaging demonstrates a fully attached retina, with no evidence of retinal detachment. B: Fundus photograph showing temporal retinal periphery demonstrating a giant retinal tear (black arrows) without subretinal fluid.

A 56-year-old woman with no known medical or ocular history presented with a 48-hour history of floaters in the right eye, without associated photopsias or visual field defects. On examination, BCVA was 10/10. The anterior segment was normal with a clear crystalline lens. Funduscopic examination identified a giant retinal tear extending from 7 to 11 o’clock in the temporal retina without evidence of subretinal fluid. The retina was fully attached, and the macula was spared (Figure 3). Examination of the left eye showed no abnormalities.

Given the absence of retinal detachment, a conservative approach was adopted with primary argon laser photocoagulation, including barrier laser retinopexy around the tear, 360° peripheral laser cerclage and prophylactic treatment of the fellow eye. Laser parameters were: spot size: 200 µm, power: 240 mW and duration: 150 ms.

**Figure 4:**
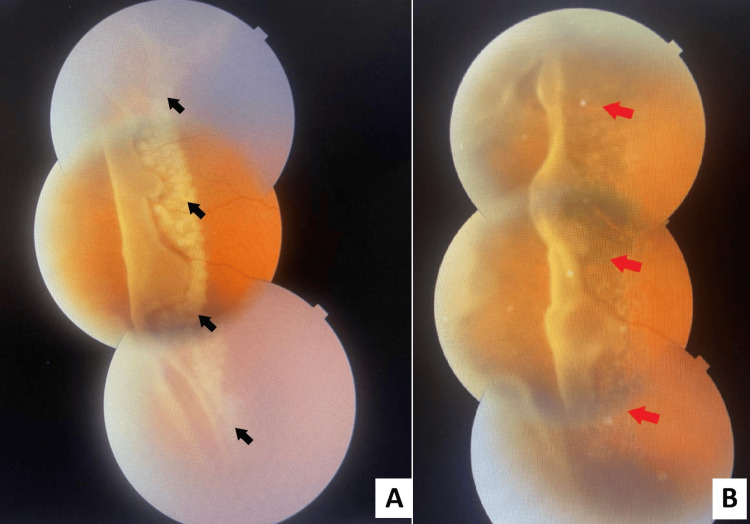
Follow-up fundus photography A: Immediate post laser fundus photography showing photocoagulation spots (black arrows). B: 18-month follow-up fundus photography showing adequate scarring (red arrows) and stable retinal tear.

At 18 months of follow-up, visual acuity remained stable at 10/10. Funduscopic examination demonstrated a flat and attached retina, with well-formed laser scars and a normal macular profile. No complications, including proliferative vitreoretinopathy, were observed (Figure 4).

## Discussion

GRT is one of the most challenging entities in vitreoretinal surgery. It’s defined as a full-thickness retinal break that extends for 90° or more in the circumferential extent, typically in the presence of a PVD [7,8]. The incidence of GRT is low, estimated between 0.094 and 0.114 per 100,000 individuals per year [4,8].

GRTs may arise from a number of preexisting conditions and risk factors including trauma (12.3%) , high myopia (25%), and hereditary vitreoretinopathies (14%) such as Marfan’s, Stickler‑Wagner, and Ehrler Danlos syndromes [1,5,6,8]. However, more than half cases remain idiopathic, highlighting the multifactorial and incompletely understood nature of this condition [7,8].

Despite their rarity, GRTs are clinically significant due to their many complications and aggressive natural history. Their strong association with rapid progression to RRD and PVR has historically justified a predominantly surgical approach [1,7]. Current standard management relies on pars plana vitrectomy (PPV), often combined with perfluorocarbon liquids (PFCL), gas or silicone oil tamponade, and sometimes scleral buckling [4,7-9]. These techniques have significantly improved outcomes, with anatomical success rates reaching up to 94%-100% in modern series [4,6]. However, surgical management is associated with a substantial risk of intra- and postoperative complications, including the creation of additional retinal breaks, retinal slippage, PVR, cataract progression, and recurrent detachment [5-7].

Traditionally, laser photocoagulation has been considered an adjunctive technique to PPV, used intraoperatively to secure the retinal edges after vitrectomy [2,9]. Its role as a primary standalone therapy remains limited, with only a small number of case reports and series described in the literature as summarized in our literature review (Table 1). Under specific conditions, laser photocoagulation alone may represent a viable alternative to surgery in selected cases.

**Table 1:** Literature review of cases of GRT without retinal detachment treated with primary laser photocoagulation Abbreviation: yo: year old; GRT: giant retinal tear; SRF: subretinal fluid; VA: visual acuity; PVR: proliferative vitreoretinopathy; ERM: epiretinal membrane.

Authors	Country	Number of cases		Age / Sex	Risk Factors	Symptoms	Tear Size & Location	Subretinal Fluid	Macular Status	Vitreous status	Management	Follow-Up	Outcome
Ton et Adam (2022) [5]	USA	1		59/Male	Prior bilateral retinal tears (laser-treated)	Blurry “black pinpoint” vision in his right eye without accompanying photopsias	6 o’clock hours (4-10 o’clock), inferior posterior GRT	No macular SRF	Macula attached	Posterior vitreous detachment	Primary indirect laser photocoagulation	4 months	Retina flat; VA improved (20/50 → 20/30); no PVR
Ao J et al. (2018) [2]	Australia	3	Case 1	74 yo/Male	Myopia, NTG, prior contralateral GRT (PPV-treated)	Floaters (2 days)	5 o’clock hours (12-5 o’clock), superotemporal	Minimal SRF	Not specified (no macular detachment reported)	Not specified	Barrier laser retinopexy	18 months	Retina flat; VA improved from 6/30 to 6/7.5
			Case 2	54 yo/Male	Myopia, lattice degeneration	Tear-shaped shadow, floaters (6 days)	4 o’clock hours, superotemporal	No significant SRF	Macula attached	Not specified	Barrier laser+additional laser+cryopexy	18 months	Retina flat; VA 6/5; mild ERM
			Case 3	48 yo/Female	Prior contralateral GRT treated with PPV	Floaters (4 days)	3-4 o’clock hours, superotemporal	No significant SRF	Not specified (no macular detachment reported)	Not specified	Barrier laser retinopexy	9 months (stable); long-term stable	Retina flat; VA improved to 6/6
Our cases (2026)	Morocco	2	Case 1	56 yo/Male	None	Blurry back pinpoint vision, photopsias in the left eye	4 o’clock hours (12 to 4 o’clock), superotemporal	No significant SRF	Macula attached	Posterior vitreous attached	Primary indirect laser photocoagulation+360° peripheral laser cerclage	12 months	Retina flat; VA conserved; no PVR
			Case 2	56 yo/Female	None	Floaters in the right eye	4 o’clock hours (7 to 11 o’clock), temporal	No significant SRF	Macula attached	Posterior vitreous attached	Primary indirect laser photocoagulation+360° peripheral laser cerclage	18 months	Retina flat; VA conserved; no PVR

Ao et al. described a small case series of three patients with GRT treated exclusively with primary laser photocoagulation, all demonstrating sustained retinal attachment over a follow-up period of 18 months [2]. Similarly, Ton and Adam reported a successful management of a posterior GRT extending up to six clock hours using laser retinopexy alone, with both anatomical stability and visual improvement maintained over time [5]. Across these reports, a consistent criterion emerges: the absence or minimal presence of subretinal fluid [2,5]. This observation is physiologically relevant, as effective laser photocoagulation requires close apposition between the neurosensory retina and the retinal pigment epithelium to induce chorioretinal adhesion. The presence of subretinal fluid disrupts this interface, reducing the efficacy of laser and allowing persistent vitreoretinal traction, ultimately leading to progression toward retinal detachment [2].

In both our patients, examination revealed the absence of subretinal fluid and a spared macula. Laser photocoagulation was applied to treat the tear and was complemented by 360° peripheral laser cerclage, along with prophylactic treatment of the other eye. Our approach differs from the previously described cases in that it aims not only to contain the tear but also stabilize the peripheral retina. This strategy is supported by the pathophysiology of GRTs, in which vitreoretinal traction is not confined to the tear margins but may involve the entire vitreous base. By applying the 360° laser, we aimed to reinforce the peripheral retinal adhesion, reduce the risk of occult or future retinal breaks, limit circumferential propagation of the tear, and provide a prophylactic barrier against delayed retinal detachment [5].

Another notable aspect of our management is the systematic prophylactic treatment of the fellow eye. Approximately 12.8% of patients develop bilateral GRT [7,10]. Although prophylactic treatment remains controversial, it can be performed to reduce the likelihood of subsequent pathology (GRT and rhegmatogenous retinal detachment) in high-risk patients [7,10].

Our findings suggest that selected GRTs without retinal detachment or significant subretinal fluid may be considered for primary laser photocoagulation in carefully chosen cases. Furthermore, our approach suggests that extending treatment to include 360° peripheral reinforcement may enhance retinal stability, although further studies are required to validate this strategy [10].

## Conclusions

Although GRTs are traditionally managed surgically because of their high risk of progression to RRD and PVR, these two cases demonstrate that GRTs without retinal detachment or significant subretinal fluid can remain anatomically stable and maintain excellent visual outcomes following primary laser photocoagulation. Together with the limited previously published literature, these findings suggest that selected GRTs identified at an early stage may be successfully managed with laser treatment alone. Early diagnosis and careful patient selection appear to be key factors for successful non-surgical management.
